# Keeping Volatile Liquids

**Published:** 1869-09

**Authors:** 


Keeping Volatile Liquids.—Chemists and others know well the difficulty of keeping very volatile liquids. Bottles of ether, for example, are shipped for India, and when they arrive are found to be more than half empty. The chemist sometimes puts a bottle of benzole or bisulphide of carbon on his shelves, and when he next requires it he finds the bottle empty and dry. The remedy with exporters is a luting of melted sulphur, which is difficult to apply and hard to remove. A new cement, therefore, which is easily prepared and applied, and which is said to prevent the escape of the most volatile liquids, will be useful information to many. It is composed simply of very finely ground litharge and concentrated glycerin, and is merely painted around the cork or stopper. It quickly dries, and becomes extremely hard, but can be easily scraped off with a knife when it is necessary to open the bottle.

